# Supplementary material for: Asynchronous magnetic resonance elastography: Shear wave speed reconstruction using noise correlation of incoherent waves
Source: Magn Reson Med. 2022 Oct 27;89(3):990–1001. doi: 10.1002/mrm.29502 (PMC9792433; doi:10.1002/mrm.29502)
Supplement: Supplementary file 1 — DATA S1 MATLAB code used to generate the numerical simulations of Figure 1 [file MRM-89-990-s002.zip › k-Wave/helpfiles/vesselFilter.html]

vesselFilter :: Functions (k-Wave)


# vesselFilter

Frangi's 3D vessel filter.

## Syntax

```
I_out = vesselFilter(I_in, grid_spacing, scales)
I_out = vesselFilter(I_in, grid_spacing, scales, ...)
```

## Description

`vesselFilter` filters a 3D image using Frangi's vessel filtering algorithm [1-3]. The algorithm works by calculating the Hessian matrix (containing second order gradients) at each image voxel. The eigenvalues of this matrix are then ordered and used to classify whether the voxel is part of a vessel. The algorithm is performed over multiple scales by convolving the input image with a Gaussian of the given input scales. The final output is taken as the maximum of the vessel filtered image across all scales.

An example of applying the vessel filter to a photoacoustic image of blood filled tubes is given below [3].

References:  
[1] A. F. Frangi, W. J. Niessen, K. L. Vincken, M. A. Viergever (1998) "Multiscale vessel enhancement filtering," MICCAI, pp. 130-137.   
[2] R. Manniesing, M.A. Viergever, W.J. Niessen (2006) "Vessel enhancing diffusion: A scale space representation of vessel structures," Med. Image Anal. 10, pp. 815-25.   
[3] T. Oruganti, J. Laufer, B. E. Treeby (2013) "Vessel filtering of photoacoustic images," Proc. of SPIE, vol. 8581, p. 85811W-1.

## Inputs

|  |  |
| --- | --- |
| `I_in` | 3D image data to filter |
| `grid_spacing` | [dx, dy, dz] in [m] |
| `scales` | array of scales to use [m] |

## Optional Inputs

Optional 'string', value pairs that may be used to modify the default computational settings.

| Input | Valid Settings | Default | Description |
| --- | --- | --- | --- |
| `'alpha'` | *(numeric scalar)* | `0.5` | Value of sensitivity parameter for metric that distinguishes between plate-like and other structures (vessel-like or ball-like). |
| `'beta'` | *(numeric scalar)* | `0.5` | Value of sensitivity parameter for metric that distinguishes between ball-like and other structures (vessel-like or plate-like). |
| `'c'` | *(numeric scalar)* | `1` | Value used to scale the sensitivity parameter for the noise metric (the sensitivity parameter itself is calculated automatically based on the magnitudes of the eigenvalues). |
| `'gamma'` | *(numeric scalar)* | `0` | Normalisation factor for scale-space derivatives. |
| `'DisplayUpdates'` | *(Boolean scalar)* | `true` | Boolean controlling whether command line updates are displayed. |
| `'Plot'` | *(Boolean scalar)* | `false` | Boolean controlling whether a maximum intensity projection of the vessel filtered image at each scale is displayed. |
| `'ColorMap'` | *(MATLAB colormap)* | `flipud(gray)` | Colour map to use if `'Plot'` is set to `true`. |

## Outputs

|  |  |
| --- | --- |
| `I_out` | vessel filtered image |

## See Also

`interpftn`, `smooth`
